# Co-infection of the respiratory epithelium, scene of complex functional interactions between viral, bacterial, and human neuraminidases

**DOI:** 10.3389/fmicb.2023.1137336

**Published:** 2023-05-04

**Authors:** Vanessa Escuret, Olivier Terrier

**Affiliations:** Centre International de Recherche en Infectiologie (CIRI)–Team VirPath, INSERM U1111, CNRS UMR 5308, ENS de Lyon, Université Claude Bernard Lyon 1, Lyon, France

**Keywords:** influenza viruses, host-pathogen interactions, bacterial superinfections, co-infection, respiratory epithelium, neuraminidase (NA), sialic acids, paramyxoviruses

## Abstract

The activity of sialic acids, known to play critical roles in biology and many pathological processes, is finely regulated by a class of enzymes called sialidases, also known as neuraminidases. These are present in mammals and many other biological systems, such as viruses and bacteria. This review focuses on the very particular situation of co-infections of the respiratory epithelium, the scene of complex functional interactions between viral, bacterial, and human neuraminidases. This intrinsically multidisciplinary topic combining structural biology, biochemistry, physiology, and the study of host-pathogen interactions, opens up exciting research perspectives that could lead to a better understanding of the mechanisms underlying virus-bacteria co-infections and their contribution to the aggravation of respiratory pathology, notably in the context of pre-existing pathological contexts. Strategies that mimic or inhibit the activity of the neuraminidases could constitute interesting treatment options for viral and bacterial infections.

## Introduction

### Sialic acids and their importance in the respiratory epithelium

Sialic acids (SA) are typically found on the extremities of glycan chains in nearly all cell types. These acidic sugars with a nine-carbon backbone are present at all cell surfaces and most secreted proteins and lipids of vertebrates and large invertebrates, mediating or modulating various normal and pathological processes (Varki, 2008; Lewis and Lewis, 2012; Schauer and Kamerling, 2018). SA are highly expressed on the surface of the epithelial cells that constitute the airways and are also major components of the secreted mucins in the respiratory tract. SA are a main source of negative charge and hydrophilicity that contribute to the rheological properties of mucus (Varki, 2008). The mucus is a complex secretion including mucins, mainly composed of carbohydrate chains. It can absorb excess water, which confers viscoelasticity and maintains hydration of the respiratory tract and mucociliary clearance (Lewis and Lewis, 2012; Zanin et al., 2016). In the airways, sialylated glycoproteins and glycolipids are strategically positioned on the plasma membranes of epithelia to regulate receptor-ligand, cell-cell, and host-pathogen interactions at the molecular level.

The structure of SA is related to their chemical link to the glycan (in particular, O-, or N-glycan), and this structure determines their biological properties. The mother molecule of the SA family is the neuraminic acid (Neu); this molecule does not occur as a free form in nature as it is immediately transformed by cyclization as an internal Schiff base. Other important SA are the N-acetylneuraminic acid (Neu5Ac), the N-glycolylneuraminic acid (Neu5Gc), and the keto-deoxy-nononic acid (Kdn) (Schauer and Kamerling, 2018). Neu5Gc is absent in humans, ferrets, seals, dogs, and most avian species (because of the absence of the enzyme cytidine monophosphate N-acetylneuraminic hydroxylase, but this SA is present in equine, bovine, and swine species). The N- and O-glycan composition of the human respiratory tract, including the nasopharynx, bronchus, and lungs, was studied by mass spectrometry, highlighting the complexity and broad spectrum of SA α2-3 and α2-6 glycans both present in the bronchus and lung tissue (Walther et al., 2013). Recently, new studies of the composition of the lung glycome revealed greater complexity with large complex N-glycans with linear poly-N-acetyllactosamine (PL) extensions terminated with α2-3 linked SA, smaller N-glycans terminated with α2-6 linked SA, and large glycosphingolipids (GSLs) glycans containing linear PL terminated with α2-3 linked SA (Jia et al., 2020).

The biology of SA is finely regulated by a class of enzymes called sialidases, also known as neuraminidases (NAs EC 3.2.1.18), that cleave the glycosidic bonds of terminal SA form carbohydrates, glycolipids, or glycoproteins (sialo-conjugates). NAs are present in a wide range of biological systems, such as viruses, bacteria, fungi, protozoa, and Mammalia (von Grafenstein et al., 2015). In this minireview, we will focus on viral, bacterial, and human neuraminidases and their functional interactions in the context of respiratory co-infections.

## Three types of Neuraminidases and their respective roles

The Carbohydrate Active Enzymes database classifies NAs into glycoside hydrolase (GH) families 33, 34, 58, and 83 ^1^ (Drula et al., 2022). The GH33 family comprises non-viral NAs, including human hydrolytic NAs (NEU1, NEU2, NEU3, and NEU4) and bacterial hydrolytic NAs, *trans*-sialidases, and anhydro-sialidases. The influenza virus NAs are classified in the GH34 family, and the *Paramyxoviridae* hemagglutinin-neuraminidases (HN) are classified in the GH83 family (Table 1). GH58 family comprises endo-N-acetyl neuraminidases (also termed endo-sialidases) found in bacteriophage K1F, for example (Stummeyer et al., 2005).

**TABLE 1:** Classification and main characteristics of viral, bacterial, and human neuraminidase.

Glycoside hydrolase family (GH)	Organism	Enzyme Name	Enzymatic activity	Structure
GH33	Human NA	NEU1 (lysosomes) NEU2 (cytosol) NEU3 (plasma membrane) NEU4 (lysosomes or mitochondria and endoplasmic reticulum)	Hydrolytic neuraminidases	monomer or oligomer
	Bacterial NA	*Streptococcus pneumoniae* (Nan A, Nan B, Nan C) Vibrio cholerae (NanH)	Hydrolytic neuraminidases (NanA, NanH) Intramolecular trans-sialidases (anhydrosialidases) (NanB)Trans-sialidases (NanC)	
GH34	Viral NA	Influenza A Virus NA (N1 to N9 Influenza B Virus NA	Hydrolytic neuraminidases	homotetramer
GH83	Viral NA	*Paramyxoviridae* family HN	Hydrolytic neuraminidases	

NA, neuraminidases; HN, hemagglutinin-neuraminidase.

The common structure of NAs is conserved up to the tertiary level with a common six-blade beta-propeller fold around a catalytic site, with several residues at similar positions in members of both families, as determined by X-ray crystallography (Taylor, 1996). Interestingly, their quaternary structures are distinct. Indeed, viral NAs are homotetramers by the assembly of the catalytic domain and need to be tetramerized to be catalytically active, while most other NAs are monomers or associate with oligomers via adjacent protein domains (Air, 2012).

### Influenza virus neuraminidases

Discovered more than 80 years ago by Hirst (1942), the NA of influenza viruses is undoubtedly the one that has been most characterized in terms of structure, activity, and biological functions. Each monomer of the influenza virus NA tetramer contains around 470 amino-acids (aa) (MW 50–60 KDa) (depending on the type of NA) and is folded from the N-terminal to the C-terminal end by a cytoplasmic tail, a transmembrane region, a stalk, and a catalytic head (Colman et al., 1983; McAuley et al., 2019). The tetrameric form allows an optimal enzymatic activity of the NA. The N2 NA is the primary reference model for numbering the residues involved in NA functionality. The catalytic site, in direct contact with the sialic acid substrate, is formed by eight highly conserved residues: three conserved Arginine residues (Arg118, Arg292, and Arg371) interact with the carboxylate group of the SA, Arg152 binds to the acetamido group on the sugar ring, and the Glu276 interacts with the 8- and 9- hydroxyl groups; the Tyr406/Glu276 is the nucleophile pair, and the Asp151 is responsible for the acid/base catalysis reaction. The catalytic site is surrounded by framework residues playing a structural role (Colman et al., 1993; McAuley et al., 2019).

The neuraminidase (NA) also has non-cleavage binding activity, mediated by a second sialic acid-binding site (2SBS) distinct from the NA catalytic site. Oseltamivir carboxylate preferentially binds to NA catalytic site, and 2SBS can attach to α2-3 sialyllactose in presence of oseltamivir carboxylate. The 2SBS site is a highly conserved feature in most avian NA (except for N3) and seems to help recruit SA substrates due to its proximity to the catalytic neuraminidase site. The 2SBS is modified or lost after adaptation from avian to another host species (the lack of conservation of the 2SBS in N1 may represent a marker of human adaptation) (McAuley et al., 2019; Du et al., 2021). Moreover, the catalytic site of the NA may also play a role in the binding to SA, as it is observed for recent human A(H3N2) viruses (Lin et al., 2010).

The different subtypes of NA are phylogenetically classified within group 1 (N1, N4, N5, and N8) and group 2 NAs (N2, N3, N6, N7, and N9); the NA of influenza B viruses form a separate group (Russell et al., 2006). The crystal structure of the different NA heads was now established for at least one representative of each subtype (Sun et al., 2014). The neuraminidase-like glycoproteins from H17N10 and H18N11 viruses isolated from bats do not contain the conserved residues involved in sialic acid binding and cleavage (García-Sastre, 2012; Li et al., 2012; Zhu et al., 2012).

Avian IAV hemagglutinin (HA) typically recognizes SA α2-3, whereas human IAV HA recognizes SA α2-6 linked to glycans. These different SA linkages have different structural conformations. In a human airway epithelium model, avian IAVs preferentially infect ciliated cells. In contrast, human IAVs infect secretory non-ciliated cells: this particular cellular tropism correlates with the pre-dominant localization of SA α2-3 or α2-6 receptors, respectively (Matrosovich et al., 2004). In human airway organoids that had similar characteristics to human airways epithelium, H1N1pdm09, H7N9, and H5N6 influenza viruses were all able to infect both ciliated cells and non-ciliated goblet cells, but not basal cells (Hui et al., 2018). When an avian IAV adapts to humans, its HA specificity evolves from α2-3 to α2-6 specificity improving human transmission; the NA specificity may evolve in the same way; however, the NA maintains a capacity for cleaving α2-3 linkages to allow virus movement toward the mucus barrier rich in α2-3 mucins (McAuley et al., 2019). Influenza strains vary in their specificity regarding the type of linkage (α2-3 or α2-6) and the type of SA residue Neu5Ac, Neu5Gc, and 9-O-Ac-Neu5Ac (Villar and Barroso, 2006).

The biological functions of NA in influenza viruses are closely related to those of the other surface glycoprotein, the HA. Both NA and HA interact with SA, and an optimal viral infection needs a balance between NA and HA activities both at the entry phase of the virus, including entry in the respiratory tract, attachment to the cell surface, and at the stage of exit after budding of viral particles (Dou et al., 2018; McAuley et al., 2019; de Vries et al., 2020).

The NA has a role in the first steps of infection (Matrosovich et al., 2004). When viral particles contained in aerosols or mucosal secretions are deposited on the mucosal surface, NA activity enables the cleavage of SA α2-3 in mucus and facilitates the access of virions to epithelial cells. At the surface of the epithelial cells, virions move to reach the base of cilia, where endocytosis is possible. By cleaving SA, NA facilitates the movement of virions on the cell surface: the NA cuts the SA and prevents the virions from rolling back. The balance of NA and HA activity drives the motility of viral particles to reach cellular sites allowing receptor-mediated endocytosis (Sakai et al., 2017; de Vries et al., 2020). Using fluorescence labeling and super-resolution microscopy, it was shown that NA glycoproteins are present in clusters at a pole of filamentous viral particles and visualized that the alternate between binding and cleaving activity causes virus directional motility away from their rich NA pole (Vahey and Fletcher, 2019).

In recent years, the entry phases of influenza viruses were better described. The different attachment routes were recently reviewed (Karakus et al., 2020; Sempere Borau and Stertz, 2021), suggesting a multivalent binding mode of virions at the cell surface and redundant ways for viral entry. The attachment of viral particles is mediated by HA and NA on sialoglycans (O-glycans, N-glycans, and Glycosphingolipid (GSL)-glycans present on cellular glycoproteins and glycolipids) and also on non-sialylated structures on phosphorylated glycans (Byrd-Leotis et al., 2019; Jia et al., 2020). The attachment and entry are also mediated by cellular co-receptors that do not bear sialoglycans: the link is rendered possible by the glycans present on the HA (Hillaire et al., 2013). After the attachment of viral particles on cells, internalization is mediated by clathrin-dependent or independent endocytosis and macropinocytosis (Karakus et al., 2020). Finally, NAs play a role in the late phases of the viral cycle enabling the efficient release of new virions by cleaving SA, which retain virions in cells and prevents the aggregation of virions between them by cleaving SA from the HAs of the virions (Air, 2012; McAuley et al., 2019).

### Parainfluenza neuraminidases

Human parainfluenza viruses (hPIV) are responsible for acute respiratory tract infections. These viruses are classified among the *Paramyxoviridae* family in two genera: hPIV1 and hPIV3 are Respirovirus, and hPIV2 and hPIV4 are Rubulavirus. These viruses bear different surface glycoproteins: the hemagglutinin-neuraminidase (HN) and the fusion protein (F). Following receptor recognition and endocytosis, the conformational change in HN activates the F protein mediating the fusion between viral and cellular membranes resulting in viral entry into the cell (Henrickson, 2003).

The global structure of HN is similar to the Influenza virus NA with a homotetramer conformation. The head domain of each monomer contains a receptor binding and a cleaving activity. Neu5Ac was identified as a receptor of HN. The role of HN was identified at the end of the viral multiplication cycle to promote the release of virions from the cells after budding. Conversely to influenza virus bearing distinct HA and NA glycoproteins, for hPIV HN both SA binding and cleaving functions reside in the same domain (Villar and Barroso, 2006).

The sequence alignment of hPIV HN and influenza NA has revealed the conservation of specific amino-acids, but framework residues are not conserved between IAV NA and hPIV HN (Colman et al., 1993). The catalytic site is formed by seven conserved aa residues (Arg192, Arg424, Arg502, Tyr530, Glu409, Glu549, and Asp216). The three Arg residues in positions 192, 424, and 502 interact with the carboxyl group of the sialic acid. The Glu409 and Glu549 interact and stabilize the Arg192 and Tyr530. The Tyr530/Glu409 is the nucleophilic pair playing a role in the catalytic reaction performed by the catalytic Asp216 residue (Chibanga et al., 2019). The crystal structure was obtained for the head domain of hPIV3 HN (Xu et al., 2013) but not yet for hPIV1 HN. The hPIV HN N-glycosylation is important in host-receptor interactions, thus, in the hemagglutinin function of HN. The presence of a 2SBS is still controversial for hPIV HN (Chibanga et al., 2019). Regarding substrate specificity, hPIV1 and hPIV3 HN preferentially recognize Neu5Ac bound to Galactose with an α2-3 linkage (Neu5Acα2-3Gal), hPIV3 also recognize Neu5Acα2-6Gal and Neu5GAcα2-3Gal (Villar and Barroso, 2006).

### Bacterial neuraminidases

Many pathogenic bacteria produce neuraminidases, most commonly associated with mucosal tissues where SA are abundant in the gut (*Vibrio cholerae*) and the respiratory epithelium (*Streptococcus pneumoniae*; *Haemophilus influenzae*; *Pseudomonas aeruginosa*). In most cases, the primary role of these bacterial NAs is to exploit SA as a carbon source or to “cap” bacterial markers such as LPS and contribute to biofilm formation (Vimr and Lichtensteiger, 2002).

One of the best-characterized examples of bacterial NAs is *S. pneumoniae*, a widespread colonizer of the nasopharynx and a major human pathogen responsible for respiratory tract infection (Shak et al., 2013). The *S. pneumoniae* genome encodes up to three distinct neuraminidases NAs (NanA, NanB, and NanC). NanA and NanB are found in most clinical isolates and are considered as pneumococcal virulence factors. NanA is present on the bacteria’s outer membrane surface and can hydrolyze α2,3- or α2,6-sialyllactoses, thus releasing α-Neu5Ac (Xu et al., 2011). NanB has a slightly different substrate specificity and acts as an intramolecular *trans*-sialidase with specificity for α2,3-sialoconjugates (Gut et al., 2008; Xu et al., 2011). NanC, found less frequently in clinical strains and the least characterized, hydrolyzes sialo-conjugated α2,3 to Neu5Ac2en (2-deoxy-2,3-dehydro-N-acetylneuraminic acid, also called DANA). This sialidase inhibitor presumably plays a regulatory role concerning the sialidase activity of NanA and NanB (Xu et al., 2011; Owen et al., 2015). Several studies suggest that the contribution of NanA to colonization and pathogenesis, observed in different *in vivo* models (Tong et al., 2000; Manco et al., 2006), relies on the role of NanA in “scavenging” host receptors to promote bacterial adhesion, the release of sialic acid as a carbon source, but also in allowing escape from the host response or altering the surface SA of other bacteria present in the same niche (Vimr and Lichtensteiger, 2002). NanB also appears to play a similar role in pathogenesis (Manco et al., 2006). Recently, *S. pneumoniae* NanB was shown to regulate NanA expression, this regulation possibly playing a role in mucus binding and mucociliary clearance (Hammond et al., 2021).

The role of NanA in the adherence and invasion of endothelial cells was demonstrated using wild type or deleted NanA mutant *S. pneumoniae* in an *in vitro* model of human brain microvascular endothelial cells and a murine model, suggesting that NanA plays an important role in the invasion of the blood-brain barrier (Uchiyama et al., 2009; Banerjee et al., 2010). The adhesin function of the N-terminal lectin part of the NanA plays a critical role in the adherence of the bacteria to endothelial cells (Uchiyama et al., 2009). The sialidase function may modify host cell receptors to reveal higher affinity ligands and increase the invasion of the endothelial cells (Uchiyama et al., 2009; Limoli et al., 2011). NanA also plays a crucial role in biofilm formation (Parker et al., 2009). The mechanism underlying this phenomenon may come from the catalytic domain able to increase sialic acids concentrations that may be used as sugars and stimulate *S. pneumoniae* growth. In addition, bacterial neuraminidases, including NanA, are also known to modulate the function of host immune factors by removing glycans which alter their stability and function [reviewed in Mathew et al. (2023)].

Similarly, to the non-enzymatic second binding site present on viral neuraminidases, a Carbohydrate Binding Module (CBM) is also present on bacterial neuraminidases, but its exact role remains to be clarified (Cantarel et al., 2009; Drula et al., 2022). This lectin domain is located in the N-terminal part, upstream of the catalytic domain of the *S. pneumoniae* NanA (Yang et al., 2015). The reconstitution of NanA structure with bioinformatics tools suggested that the lectin and catalytic domains were separated with a 16 amino-acid long flexible linker, whereas these two domains for NanB and NanC formed rigid globules stabilized by multiple interdomain interactions (Sharapova et al., 2018).

The opportunistic pathogenic bacterium *P. aeruginosa*, one of the primary etiological agents of pulmonary infections in cystic fibrosis patients (CF), is a particular case, as this bacterium does not use SA as a carbon source and does not possess sialic acid conjugated LPS (YuA et al., 1988). The comparative infection study in a mouse model of wild-type strains versus a mutant strain of *P. aeruginosa* deleted from its NA locus (D2794) showed the role of bacterial NA in biofilm formation, highlighting its role in virulence (Soong et al., 2006). Neuraminidase appears to play a role in the pathogenesis of *P. aeruginosa* in the context of cystic fibrosis, with NanA expression being increased under hyperosmolarity conditions, under the control of the gene involved in alginate expression and in the mucoid phenotype (Cacalano et al., 1992), which would further increase bacterial adhesion.

### Mammalian neuraminidases

Four NAs have been identified in mammals (NEU1, 2, 3, and 4), exhibiting different localizations and functions (Monti et al., 2010; Miyagi and Yamaguchi, 2012). NEU1 is localized in lysosomes; its lysosomal activity is closely associated with cathepsin A and beta-Galactosidase. Mutations in the human NEU1 locus led to the lysosomal storage disease called sialidosis. NEU2 is mainly localized in the cytosol, but like NEU1, it can also be translocated to the membrane via mechanisms that are still poorly characterized to play other roles, particularly in the immune response (van der Wal et al., 2020). NEU3 is located in the plasma membrane, particularly on the surface of certain epithelia, and NEU4 is located in mitochondria. Some functions of mammalian NAs seem to be in opposition or complementary, e.g., NEU3 and NEU4 oppositely regulate neuronal differentiation (Miyagi et al., 2018), and NEU1 and NEU3 target glycoproteins and gangliosides, respectively, Monti et al. (2010), Miyagi and Yamaguchi (2012). Interestingly, relevant to the focus of this review, these NAs, most notably NEU1 and NEU3, have been shown to have activity at the respiratory epithelium, contributing to 70 and 30% of the detected sialidase activity, respectively (Lillehoj et al., 2014, 2015).

## Functional interactions between the different neuraminidases in the context of respiratory epithelial viral and bacterial infections

**FIGURE 1:**
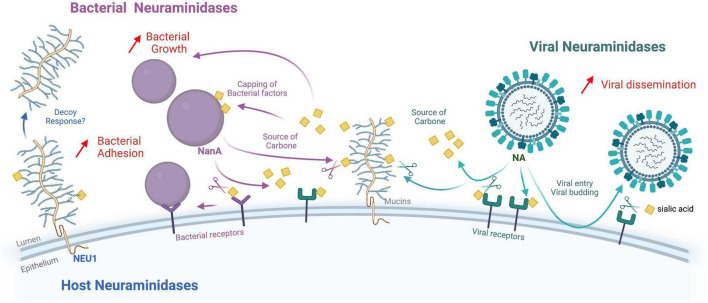
Functional interactions between viral, bacterial, and human neuraminidases. This diagram summarizes the known functions of the different neuraminidases (NA) in the context of coinfections (Viral, bacterial and host neuraminidases are pictured in green, purple, and blue, respectively. Sialic acids are represented by yellow squares). While viral NA (NA/HN) play a crucial role in virus entry and budding mechanisms, bacterial NA (NanA, NanB, and NanC) allow the exploitation of sialic acids as a carbon source or to “cap” bacterial markers such as LPS, and/or promote biofilm formation. The role of host neuraminidases at the epithelial level remains relatively uncharacterized, but their action on glycoproteins and gangliosides certainly plays an important role in regulating different types of cellular responses. Viral NA can promote bacterial infections by “stripping” SA that mask bacterial receptors on the surface of the epithelium. Another mechanism that may explain the frequent superinfection by bacteria after influenza virus infection is that influenza NA increases the availability of sialic acid as a nutrient for bacteria (carbon source). Influenza NA and bacterial NA play synergistic roles in upper respiratory tract superinfections. Host neuraminidase NEU1 activity reduces EGFR stimulation by EGF and increases *P. aeruginosa* adhesion through increased MUC1 binding to bacterial flagellin. Interestingly, other work has confirmed that *P. aeruginosa* mobilizes NEU1 via its flagellin to enhance its pathogenicity. In response, the cell releases the MUC1 ectodomain into the airway lumen as a hyperadhesive decoy receptor. Figure created with Biorender.com.

Within the complexity of the respiratory epithelial tract, the interactions between viral and bacterial pathogens may lead to more severe diseases than those caused by viruses or bacteria alone. The mechanisms involved in viral and bacterial co-infections or superinfections were reviewed recently (Oliva and Terrier, 2021). Lesions of the respiratory epithelium, by direct viral cytopathic effect or inflammatory response, expose the basement membrane and favor bacterial adherence to epithelial cells; lesions also decrease mucociliary clearance. The viral infection may also induce immunosuppression and leukopenia that favor bacterial superinfections. In this review, we will focus only on data regarding mechanisms implying the different NAs, summarized in Figure 1.

### Viral NAs and bacterial infections

Viral NA can promote bacterial infections by “stripping” SA that masks receptors on the epithelium’s surface. Experimental studies have shown that Influenza virus NA or hPIV HN can remove SA on host cells and expose *S. pneumoniae* receptors (Peltola and McCullers, 2004). The mechanisms by which Influenza NA increases bacterial adherence are not fully understood, but different hypotheses are suggested. Influenza NA, through the removal of SA from latent TGF-β, was demonstrated to activate TGF-β expression (Carlson et al., 2010), which in turn up-regulates host adhesion molecules, fibronectin, and α5-integrin, increasing the bacterial binding of group A *Streptococcus* (Li et al., 2015). Epidemiological studies have shown an association between influenza and *Neisseria meningitidis* infections; one hypothesis is that influenza infection contributes to a higher adhesion of *N. meningitidis* to respiratory epithelial cells, thus permitting entry through the epithelial barrier to the blood and meningeal spaces. Indeed, a recombinant influenza NA increased the adhesion of *N. meningitidis* strains serogroups B, C, and W135 to Hec-1-B human epithelial cells. The influenza NA permits the cleavage of sialic acid on capsular polysaccharides; this could unmask subcapsular bacterial factors that could interact with cell membrane receptors (Rameix-Welti et al., 2009).

The mechanism that may explain the frequent superinfection by *S. pneumoniae* after Influenza virus infection is that influenza NA will increase sialic acid availability as nutrient for *S. pneumoniae* promoting its proliferation. Consequently, suppressing mucins or using mucolytic treatment limits influenza-promoted *S. pneumoniae* replication (Siegel et al., 2014). In addition to influenza virus, there is also a direct link between hPIV infection and *S. pneumoniae* infections in young children (Grijalva et al., 2014). Neuraminidase inhibitors have an impact on the reduction of influenza-related complications and their use may reduce the use of antibiotics. This impact may be due to the fact that blocking influenza NA will limit sialic acid availability and limit bacterial superinfections (McCullers, 2014). A similar impact of the use of hPIV HN inhibitors may limit *S. pneumoniae* superinfections (Alymova et al., 2005, 2009).

The NA of each pathogen may also mediate pathogen interactions, and interactions may be possible via the ubiquitous SA. Visualized by confocal and super-resolution microscopy, the direct interaction between IAV and different bacteria (*S. pneumoniae*, *S. aureus*, and *H. influenzae*) may promote bacterial adherence (Rowe et al., 2019). More recently, a study has shown that hPIV3 can recognize the α2-3 linked SA on capsular polysaccharides of Group B *streptococci*. Experiments of co-infections delayed hPIV3 infection but increased Group B *streptococci* adherence to virus-infected Hep-2 cells (Tong et al., 2018).

### Impact of bacterial NAs on viral infection

In the Human airway epithelial model (HAE), NAs with different SA cleavage specificity were used to better understand the physiopathology of viral infections. Non-ciliated cells present a higher proportion of α2-6-linked SA, while ciliated cells possess both α2-3 and α2-6-linked SA. Experimental studies on HAE have shown that avian IAV and hPIV3 mainly infect ciliated cells, whereas human IAV mainly infects non-ciliated cells (Matrosovich et al., 2004; Zhang et al., 2005; Thompson et al., 2006). Treatment of HAE with the *Vibrio cholerae* NA, which cleaves SA residue with α2-3, α2-6, or α2-8 linkages, did not significantly impact human IAV infection but abolished hPIV3 infection. These results suggest that SA are more important for hPIV3 infection than for human IAV infection of HAE (Thompson et al., 2006). The use of NAs with different cleavage specificity provided evidence that hPIV3 utilizes α2-6-linked SA on ciliated cells to initiate infection (Zhang et al., 2005). The IAV NA and bacterial NA play synergistic roles in upper respiratory tract superinfections. The role of the bacterial NA was recently studied using different models. In a mouse model, NanA deficient *S. pneumoniae* were impaired in their ability to induce nasal and middle ear infections. IAV and NanA synergize to influence bacterial pathogenesis (Wren et al., 2017).

### Interactions between mammalian NEUs and pathogens

Mammalian NAs and their interactions with pathogens have been relatively less studied than their counterparts in bacteria and viruses. Nevertheless, several studies have highlighted the role played by NEU1 in the adhesion and pathogenicity mechanisms of certain bacteria. Indeed, both in the A549 cell line and in primary respiratory epithelial cells, it has been demonstrated that two receptors present on the surface of respiratory epithelia, Epithelial Growth Factor Receptor (EGFR) and mucin 1 (MUC1), are substrates of NEU1 *in vivo*. NEU1 regulates the activation of signaling pathways associated with these receptors, in particular, the ERK1/2 (extracellular signal-regulated kinase) pathway. NEU1 activity reduces EGFR stimulation by EGF and increases *P. aeruginosa* adhesion via increased binding of MUC1 to bacterial flagellin (Lillehoj et al., 2012). Interestingly, other work has confirmed that *P. aeruginosa*, mobilizes NEU1 via its flagellin to increase its pathogenicity. Still, in response, the cell releases the ectodomain of MUC1 into the airway lumen as a hyper-adhesive decoy receptor (Lillehoj et al., 2015). Because of its localization to the surface of epithelia and ciliated brush borders, and its ability to desialylate surface gangliosides, NEU3 could play a role similar to that of NEU1, but this has yet to be demonstrated.

## Treatment options and research avenues inhibiting or using neuraminidases in the context of viral and bacterial infections

Sialic acids and NAs are ubiquitous molecules that mediate cell infections by viral and bacterial pathogens and contribute to increasing their pathogenicity. Therefore, there may be an interest in inhibiting viral and bacterial NAs to prevent viral and bacterial propagation or cleaving SA to prevent viral infections.

### Neuraminidase inhibitors with anti-influenza activity

The discovery of the Neu5Ac2en (DANA) initiated the discovery of neuraminidase inhibitors (NAIs). Knowledge of the structure of the active site of NA interacting with SA or DANA has led to the discovery of NAIs with antiviral activity (Colman et al., 1983; Varghese et al., 1983). Zanamivir, very close to DANA (4-guanidino-Neu5Ac2en), is only active by inhalation. Oseltamivir carboxylate contains a cyclohexene base and a bulky hydrophobic group (6-pentyl ether chain): it can bind to the active site of the viral NA after reorientation of the E276 residue allowing the formation of a hydrophobic pocket (Varghese et al., 1998). Peramivir bears a cyclopentane base, a 4-guanidino group, and a hydrophobic side chain (Babu et al., 2000); due to low oral bioavailability, it is used parenterally for severe infections in adults in the USA, Japan, and Korea (McLaughlin et al., 2015). Laninamivir is a long-acting NAI that contains a 4-guanidino group and a 7-methoxy group. The prodrug laninamivir octanoate used by inhalation is converted to laninamivir in the lungs with a prolonged duration of action; it is approved for use in Japan in uncomplicated forms (Watanabe et al., 2010). The trials concerning the efficacy of oseltamivir in the curative treatment of influenza show a limited clinical efficacy (Hayden et al., 1999; Nicholson et al., 2000; Treanor et al., 2000). Meta-analyses of randomized clinical trials have demonstrated that oral oseltamivir, in the context of uncomplicated influenza, decreases the duration of clinical signs by 1 day and increases the risk of nausea (Jefferson et al., 2014; Dobson et al., 2015). Treatment with oseltamivir reduces respiratory complications requiring antibiotics and hospitalizations (Dobson et al., 2015). Meta-analyses of individual data (compilation of 78 observational studies on 29,234 patients of all ages between 2009 and 2011) show a 50% reduction in mortality when taking NAIs less than 48 h after the onset of signs clinics (Muthuri et al., 2014). The addition of oseltamivir carboxylate at 1 μM on HAE culture and to IAV suspensions used for infection decreased significantly (20 to 500-fold fewer cells) the number of infected cells compared to untreated cultures. These results indicate that inhibition of the IAV NA suppresses the initiation of the IAV infection (Matrosovich et al., 2004). The potential inhibition by these NAIs of NAs other than IAV NAs represent one research avenue to discover other molecules.

### Neuraminidase inhibitors with anti-hPIV HN activity

The roles of hPIV HN in the viral multiplication cycle at early steps (attachment to SA and activation of the F protein for fusion) and later stages (release of new viral particles by the neuraminidase catalytic site) make it an ideal target for antiviral agents. Competitive inhibitors derived from DANA were studied as possible hPIV HN inhibitors. The *in vitro* zanamivir IC_50_ against hPIV HN was very high (evaluated at 0,25mM). Using the crystal structure of Newcastle Disease virus (NDV) HN, BCX 2798 and BCX 2855 inhibitors were developed. The hPIV inhibitor BCX-2798 was the most studied compound presenting prophylactic efficacy in a mouse model of hPIV1 infection (Alymova et al., 2009). Therapeutic options for inhibition of hPIV HN with DANA-derived analogs were reviewed recently, and targeting the receptor binding activity appears more efficient than blocking the neuraminidase activity alone (Chibanga et al., 2019).

### Neuraminidase inhibitors to elucidate mammalian NAs activity

Studies using recombinant purified mammalian NAs (NEU) and the MUNANA (4’-methylumbelliferyl α-d-N-acetylneuraminic acid) substrate revealed a low inhibitory activity of DANA, mainly against NEU3 and NEU4, and of zanamivir against NEU2 and NEU3 (Hata et al., 2008; Richards et al., 2018). Recent studies reported the discovery of NEU1, NEU2, and NEU3-specific inhibitors derived from DANA (Guo et al., 2018a,b). These specific inhibitor molecules are interesting tools to elucidate mammalian NAs roles, and a recent study has shown that NEU1 and NEU3 promote leukocyte infiltration, whereas NEU4 was anti-inflammatory (Howlader et al., 2022).

### Neuraminidase inhibitors and *S. pneumoniae* NanA and NanB

Crystal structures of *S. pneumoniae* NanA in complex with zanamivir or oseltamivir carboxylate revealed a weak to medium NanA competitive inhibition, respectively (Gut et al., 2011). Other molecules katsumadain A and artocarpin, can inhibit recombinant NanA (rNanA) at low micromolar concentrations in different NA inhibition assays; however, artocarpin was the only one able to inhibit *S. pneumoniae* adherence to A549 cells, reduce biofilm formation and bacterial growth (Walther et al., 2015). The study of S. *pneumoniae* NanA has revealed the evolutionary diversity of this enzyme and the different inhibitory efficiency of oseltamivir and DANA on these enzymes (Xu et al., 2016). The potential role of *S. pneumoniae* NanA and NanB in post-influenza virus infection complications was studied in A549 and MDCK cells. The addition of low dilutions (less than 1:1,000 and 1:100) of rNanA or rNanB hampered the virus spread in A549 cells, whereas higher dilutions (more than 1:10,000 and 1:1,000) promoted virus spread. These results suggest that high levels of rNanA or rNanB remove viral receptors and prevent cells from IAV infection, whereas at higher concentrations, rNanA, and B enhance the cleavage function for viral release (Walther et al., 2016). In this co-infection *in vitro* model, the presence of zanamivir at 1 μM inhibited the IAV NA, but zanamivir was inactive on rNanA, which could promote the viral release and spread. Oseltamivir at 1 μM inhibited both IAV NA and rNanA, and viral spread was limited (Walther et al., 2016). These results suggest that the discovery of molecules able to inhibit both viral NAs and bacterial NAs may prevent complications linked to viral and bacterial co-infections.

### Use of neuraminidases to cleave sialic acids and prevent viral infections

The DAS181 is a recombinant fusion protein composed of *Actinomyces viscosus* sialidase catalytic domain fused to an epithelium anchorage heparin-binding domain (Malakhov et al., 2006). The DAS181 removes α2-6 or α2-3 linked to terminal galactose SA from respiratory epithelium cells, preventing the infection by viruses using sialic acid as a receptor (Malakhov et al., 2006). Influenza and hPIV infections can be controlled *in vitro* and *in vivo* by the DAS181 (Fludase^®^) used topically as an inhaled treatment. Studies performed in a mouse model confirmed that DAS181 was efficient for preventing and treating influenza A(H5N1) and oseltamivir-sensitive or resistant A(H7N9) infection in a mouse model (Belser et al., 2007; Marjuki et al., 2014). A phase II clinical trial performed in healthy adult participants has shown that the DAS181 allows a significant decrease in influenza viral load. However, no impact was detected on alleviating clinical symptoms, possibly due to the healthy patient recruitment (Moss et al., 2012). A phase II clinical trial (NCT04298060) is registered to study the DAS181 in patients with severe influenza virus infections. DAS181 was also used as a compassionate treatment in immunocompromised patients infected with hPIV. The results of phase 2 clinical trial NCT01644877 studying the clinical impact of DAS181 in immunocompromised patients suffering from a low respiratory infection with hPIV suggest that DAS181 improves oxygenation in hPIV infected immunocompromised patients not requiring mechanical ventilation (Chemaly et al., 2021). Albeit these encouraging data, the therapeutic use of DAS181 has some limitations. The DAS181 is well tolerated, although some possible hepatic disturbances (Moss et al., 2012). Antibodies directed against DAS181 develop in treated patients; this could preclude a prolonged or repeated use of this molecule (Zenilman et al., 2015). SA and mucins function as decoys for pathogens and are essential to protect epithelial cells. The desialylation of the epithelium may expose host receptors and increase bacterial adherence and superinfection (Zhang, 2008). However, bacterial adherence needs exposure of basal membranes after epithelial necrosis mediated by viral infection (Nicholls et al., 2013). DAS181 treatment alone did not result in any cytopathic HAE effect and did not increase the adhesion of *S. pneumoniae* (Nicholls et al., 2008).

The Carbohydrate Binding Module (CBM) from *S. pneumoniae* has been used as a preventive strategy, as a single intranasal administration 7 days before challenge, to mask sialic acids and protect mice from a lethal challenge by A(H1N1)pdm09 influenza virus (Connaris et al., 2014). The idea of diverting the natural biological properties of some pathogens to mask host receptors and prevent infection by another pathogen is an is an interesting avenue for development.

## Conclusion

Today, we have a relatively good understanding of the central role of sialic acids and neuraminidases in pathogen biology. In comparison, our knowledge of the role of different neuraminidases in the mutual interactions between viruses, bacteria, and host cells is still limited and deserves further exploration in the future. In the context of respiratory co-infections between viruses and bacteria, the respective contribution of host neuraminidases, virus(es), and bacteria to the evolution of respiratory pathology remains to be explored and better understood. This will require the development of new biologically relevant experimental models that allow a comprehensive approach to the study of host-pathogen interactions, requiring a combination of techniques from different disciplines. These different sialidases with similar enzymatic activities could constitute a common target of interest for the future development of specific treatments for bacterial superinfections, for example.

## Author contributions

VE and OT wrote the first draft of the manuscript and wrote the sections of the manuscript. Both authors contributed to manuscript revision, read, and approved the submitted version.

## Footnotes

1
http://www.cazy.org
